# Isolated Langerhans cell histiocytosis in the hypothalamic-pituitary region: a case report

**DOI:** 10.1186/s12902-019-0474-0

**Published:** 2019-12-19

**Authors:** Weibin Zhou, Jia Rao, Chengjiang Li

**Affiliations:** Department of Endocrinology, the First Affiliated Hospital, College of Medicine, Zhejiang University, Hangzhou, 310003 Zhejiang China

**Keywords:** Langerhans cell histiocytosis, Pituitary stalk thickening, Isolated hypothalamic-pituitary lesion, Central diabetes insipidus

## Abstract

**Background:**

Langerhans cell histiocytosis (LCH) is a rare disease that mainly affects children, but this disease is significantly rarer in patients who are older than 15 years. In this disease, any organ can be involved. The skeleton, skin and lung are commonly affected, and isolated hypothalamic-pituitary (HP) involvement is relatively rare. Here we report a 17-year-old adolescent with isolated HP-LCH of enlarged pituitary stalk presented with central diabetes insipidus (CDI).

**Case presentation:**

A 17-year-old male adolescent with polydipsia and polyuria accompanied with elevated serum sodium level and low urine osmolality for 3 weeks was referred to our hospital. After admission, hormonal evaluation showed that his growth hormone (GH) was slightly elevated, and serum osmolality and glucose were normal. The fluid deprivation-vasopressin test demonstrated CDI. Imaging examination showed an obvious thickening of the pituitary stalk. Lymphocytic hypophysitis, sarcoidosis and granulation tissue lesions were suspected. After oral 1-deamino-8-Darginine vasopressin (DDAVP) and prednisone were administered for 2 months, symptoms were relieved, and he discontinued taking the drugs by himself. On reexamination, imaging revealed changes in the size and shape of the pituitary stalk, with thickened nodules. Then, a diagnostic biopsy of the pituitary stalk lesion was performed. Immunohistochemistry confirmed the definitive diagnosis of LCH. The clinical symptoms subsided with oral hormone replacements.

**Conclusion:**

CDI is a rare symptom in children and adolescents. Most of the causes are idiopathic, while others are caused by central nervous system (CNS) disorders. Meanwhile, lymphocytic hypophysitis, germinoma, LCH and other CNS disorders can all present as thickening of the pituitary stalk, diffuse enlargement of the pituitary gland, and weakening of high signal intensity in the neurohypophysis on magnetic resonance imaging (MRI). The differential diagnosis among these diseases depends on immunohistochemistry evidence.

## Background

Langerhans cell histiocytosis (LCH) is a rare disease of the monocyte- macrophage system characterized by clonal proliferation of epidermal dendritic cells [1–3]. The pathogenesis of this disease is not clear. Although the cell benign morphology of LCH is relatively similar to an inflammatory disease, its clonal proliferation pattern is an important signal of neoplasia [3]. Single or multiple lesions composed of the proliferative Langerhans cell lead to clinical manifestations ranging from single organ lesion to widespread multiple organ involvement [1, 4]. The skeleton (78.7%), and skin (36.7%) are the most frequently involved organs. The involvement of multiple organs such as the skin, lymph node, lung, spleen, liver, bone marrow, ear, nose and throat demonstrates a poor prognosis [5]. Therefore, it is essential to establish the diagnosis as early as possible.

Hypothalamic-pituitary region (HPR) infiltration is present in 5–50% of children with LCH [6]. Central diabetes insipidus (CDI) and anterior pituitary deficiencies (APD) frequently develop in patients with HPR- involved LCH. The most common type of hormone deficiency in children and adults with HPR involved LCH is growth hormone (GH) deficiency (53–67%) [7] followed by gonadotropin (53–58%) and thyroid-stimulating hormone (3.9%) deficiencies [6, 8, 9]. A single-center study [10] recording all patients with isolated HPR involved LCH from 2007 to 2015 indicated that all cases had CDI as the earliest symptom. And some of the patients also had APD. However, APD can also be seen as the consequences of surgery, radiotherapy or chemotherapy [11].

Huo et al. summarized the magnetic resonance imaging (MRI) features of patients with HPR involved LCH as the following: 1) a single lesion on the HPR and involvement of the pituitary stalk; 2) isointensity signals on both T1-weighted image (T1WI) and T2-weighted image (T2WI) in most cases; 3) homogeneous enhancement in most cases; and 4) relatively clear borders in most cases [10]. However, it is still hard to differentiate HPR involved LCH from other diseases, such as germinoma or lymphocytic hypophysitis (LYH) by MRI.

As a rare disease, LYH is the most common chronic pituitary gland inflammation. APD with CDI and growth retardation are the most significant clinical manifestations in children with LYH [12]. The gold standard for diagnosis of LYH relies on histopathological examination. Central nervous system germ cell tumors (CNS-GCTs) represent a rare heterogeneous group, and they are usually located in the pineal and suprasellar regions. The clinical manifestation frequently includes endocrine disorders, while tumor markers or human chorionic gonadotropin (HCG) of CNS-GCT patients may be elevated [13].

Isolated HP-LCH is a rare, elusive disease that may be easily misdiagnosed. Therefore, this case report aimed to review the characteristic of isolated HPR involved LCH and to provide clinical experience for reducing the misdiagnosis rate.

### Case presentations

A 17-year-old male adolescent presented to our hospital with polydipsia and polyuria for 3 weeks. He was found to have hypernatremia with a sodium level of 150.4 mmol/L (normal 135–145) and low urine osmolality in outpatient laboratory tests. Prior to admission, there was no history of craniocerebral trauma or surgery, headache, nausea, vomiting, hyperpyrexia, visual field defect and other symptoms. He denied taking any toxic food or lithium drugs. His medical history was unremarkable and there were no similar patients in his family.

**Fig. 1:**
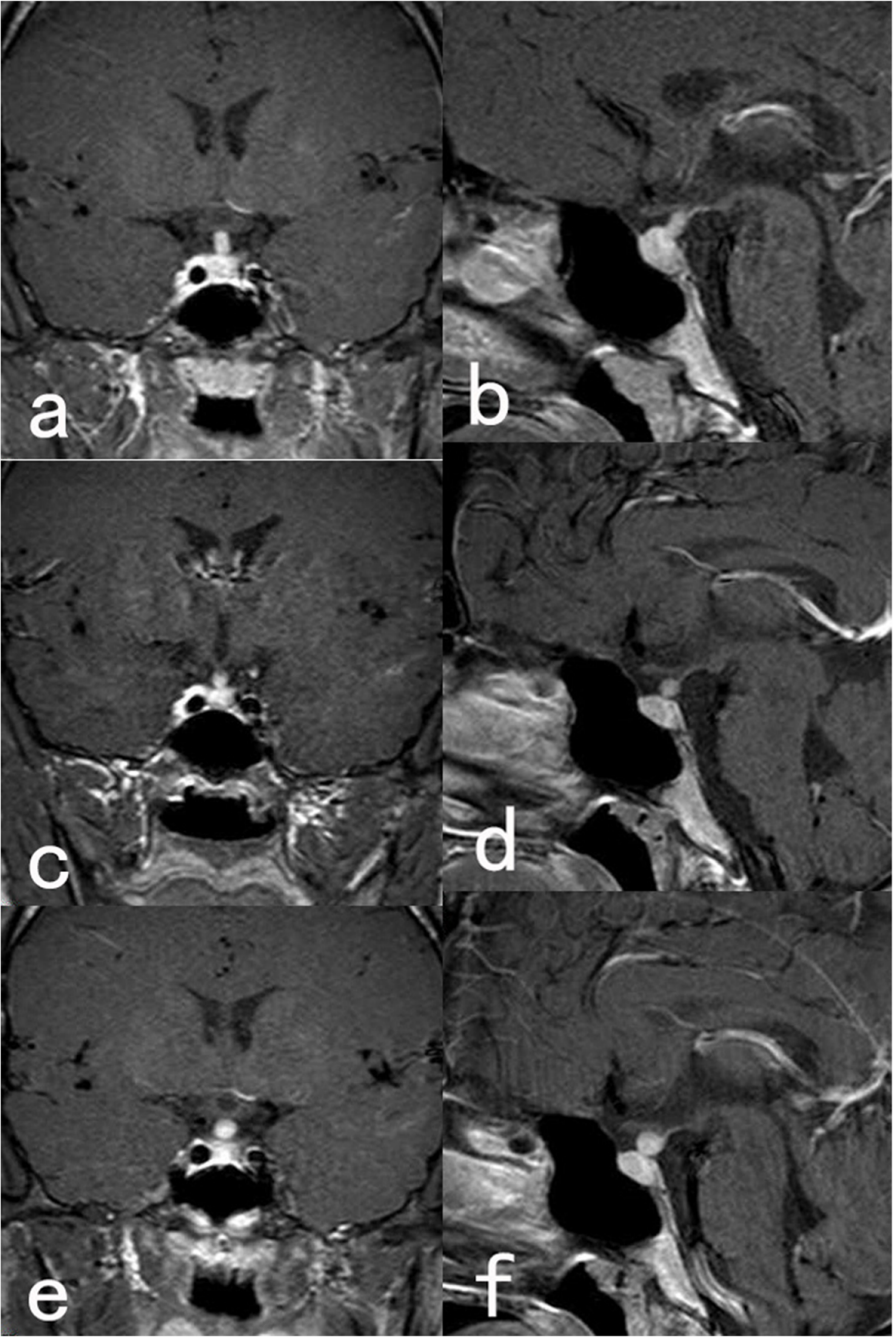
**a** Coronal and **b** sagittal magnetic resonance imaging (MRI) (T1-weighted with gadolinium enhancement) demonstrating an enlarged pituitary stalk and the neurohypophysis hypersignal deficiency. **c** Coronal and **d** sagittal T1WI showing the pituitary stalk is a nodular thickening with both size and shape have been changed after the patient discontinued the treatment for 1 month. **e** Coronal and **f** sagittal T1WI showing the nodular-thickening pituitary stalk is continuous enlarging after the patient continued the oral drugs for 2 months

Physical examination was all normal. His 24-h urine volume and 24-h drinking water volume were 13,000 ml and 10,500 ml, respectively. Hormonal measurements showed a slight increase in his GH level (17.90 ng/ml, reference value 0–8), which could be suppressed by the high-sugar-inhibition test. The patient’s circadian cortisol rhythm was regular. Other hormone levels were within the normal ranges. Chest computed tomography (CT), brain and skull CT, and thyroid gland and abdominal ultrasound revealed normal findings. The result of fluid deprivation-vasopressin test demonstrated CDI. Subsequently, enhanced pituitary MRI was performed, and it showed an obvious thickening of the pituitary stalk, involving the funnel structure, and the enhancement of the posterior pituitary gland was obvious and uniform (Fig. 1a, b).

Lymphocytic hypophysitis, sarcoidosis, and granulation tissue lesion were suspected. He was empirically administered with oral 1-deamino-8-D-arginine vasopressin (DDAVP) and prednisone and the symptoms improved.

Two months after the patient’s complaints, he discontinued the treatment by himself. And the reexamination of enhanced pituitary MRI revealed changes in the size and shape of the pituitary gland with a nodular thickening of the pituitary stalk (4.5*5.0 mm), which appeared to be moderately homogeneous enhanced. The optic chiasma was normal, and the morphology and signal of the rest of the hypothalamus were not different (Fig. 1c, d).

The plasma HCG, follicle-stimulating hormone, luteinizing hormone, adrenocorticotropic hormone, cortisol, GH, prolactin, insulin-like growth factor-1, thyroid hormones, alpha fetoprotein and lactate dehydrogenase were all within the normal ranges, except for a slightly increased estradiol (46.5 pg/ml, reference value 0–44.5). The biochemistry, albumin, cell counts, and immunoglobulin G (IgG) of cerebrospinal fluid were normal and bacterial culture was negative. The intracranial pressure was 105 mmH2O. Therefore, germinoma was nearly excluded from the differential diagnoses.

**Fig. 2:**
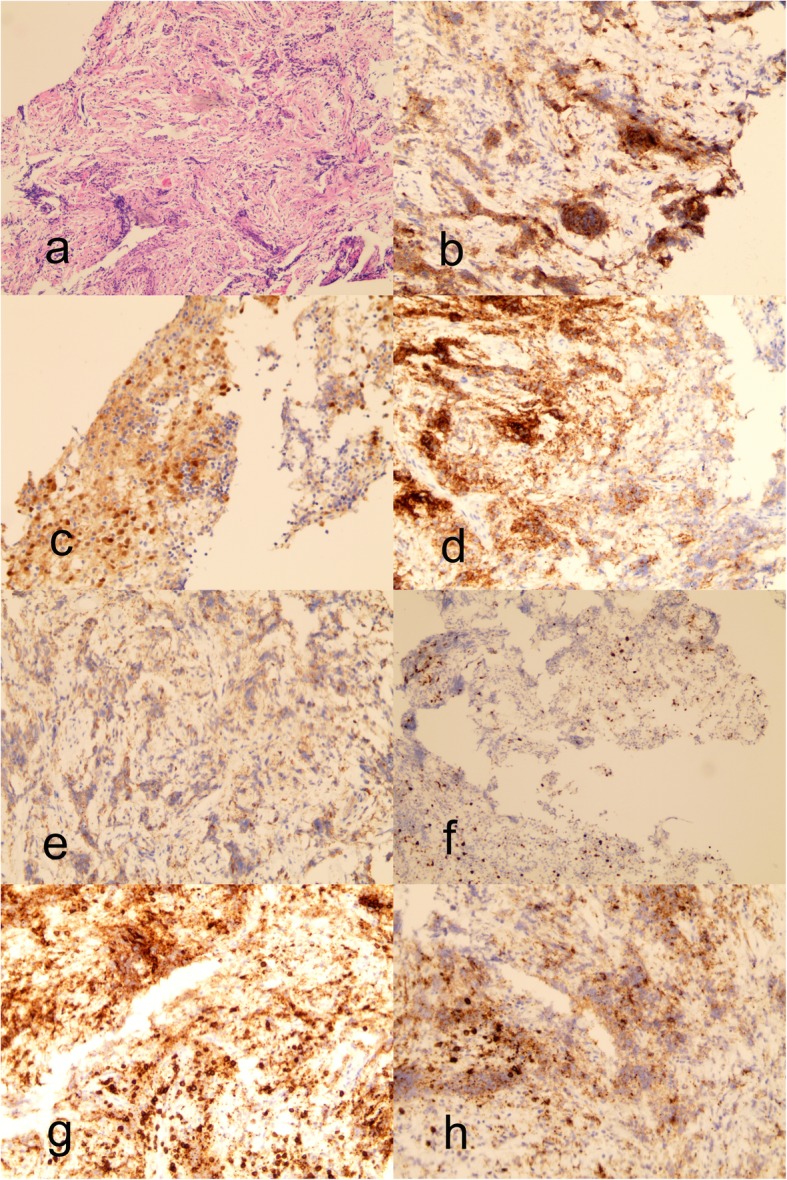
**a** Lesion histopathological examination of the pituitary stalk: hematoxylin and eosin staining (HE× 100). **b-h** Immunohistochemical examination (SP × 200). **b** Langerhans’ cells (+), **c** S-100 (+), **d** CD1a (+), **e** CD68 (+), **f** ki67 (+), **g** CD3 (+), **h** CD20 (+)

Based on the clinical characteristics, hormone measurements and imaging changes, sarcoidosis and granulation tissue lesions were suspected. DDAVP was administered again to the patient since he denied pituitary biopsy. After two more months, reexamination of enhanced MRI showed that the tubercle of the pituitary stalk was larger compared with that in the previous MRI (Fig. 1e, f). At last, the patient underwent a diagnostic biopsy of the pituitary stalk in our hospital. The intraoperative pathology found a small piece of tissue with inflammatory cell infiltration and lymphocyte proliferation. The immunohistochemistry of the biopsy specimen showed that the lymphohistiocytic infiltration was positive for Langerin (Fig. 2b), S^− 100^ protein (Fig. 2c), cluster of differentiation (CD) 1a (Fig. 2d), CD68 (Fig. 2e), ki67 (Fig. 2f), CD3 (Fig. 2g), and CD20 (Fig. 2h) while negative for IgG4, placental alkaline phosphatase (PLAP), octamer-binding transcription factor 4 (OCT-4), keratin (CK), steroidogenic factor 1 (SF1), Tpit, and Pit-1. Thus, the patient was finally diagnosed with CNS-LCH. The patient recovered well from surgery, with central adrenocortical insufficiency, CDI and hypothyroidism. Postoperative CT and MRI showed postoperative changes in the pituitary stalk. Oral DDAVP, hydrocortisone, and levothyroxine were given. As suggested by a hematologist, no chemotherapy or radiation was recommended during watchful observation since single- site LCH frequently has a good prognosis. After discharged, he received regular follow-up and so far, no local reoccurrence was noticed.

## Discussion and conclusion

LCH is a rare disorder that can be observed in all age groups, mainly affecting the pediatric population. The estimated annual incidence of LCH is approximately 6:100000 to 10:100000, and this disease is significantly rarer in populations aged > 15 years (1:100000) [14]. Among the patients, the male/female ratio was about 1.8.

LCH is characterized by abnormal clonal proliferation and accumulation of antigen-presenting dendritic cells at multiple tissues and organs with a wide range of clinical manifestations and histologic presentations [14]. Because of the benign morphology of Langerhans cells, LCH was not considered as a neoplasm in the past. However, as recurrent oncogenic BRAF V600E mutations were identified in LCH, it is considered as a neoplastic disease that may respond to RAF pathway inhibitors [3, 15]. LCH often involves multiple organs especially in the extra-cranial sites. The classification of LCH includes three stages: first, a single system disease with good prognosis; second, multi-system disease; and finally, multi-system disease of organ dysfunction with the worst prognosis [16].

Our patient was diagnosed with LCH isolated to the HPR, which is relatively rare. In a large series of patients with LCH (up to 1242 study subjects), only 0.04 to 0.6% had HPR involvement without extracranial lesions. In 2010, the French national LCH registry had enrolled 1236 LCH patients under 18 years old. During follow-up, only 4 of the 1236 LCH patients (0.32%) were still remained isolated HP-LCH after 5.1 to 10 years [17]. In Howarth’s retrospective study of 314 patients with LCH recruited during a 50-year period, only 2 of the 314 LCH patients (0.6%) had isolated HP-LCH, while all patients had CDI [6, 11]. In Marchand’s study [18], a 2-year follow-up was taken on 14 LCH patients with an enlarged pituitary stalk, the MRI showed some stalk volumes increased, some decreased and others remained stable. Another study showed a 5-year follow-up of a LCH patient whose hypothalamic lesion (involving the mammillary bodies and a slightly thickened pituitary stalk) was gradually regressing after persistently hormone replacement therapy [4]. Isolated HPR lesion is a clinical challenge because neither clinical manifestations nor MRI can make a definitive diagnosis, and the risk of obtaining a diagnostic biopsy remains high. However, LCH patients with single-system and single site often carry a good prognosis. Nagasaki et al. reported a case of 13-year-old girl whose solitary neurohypophyseal LCH could shrink spontaneously up to near remission level. And after three years follow-up, no de novo lesions had been found [19]. In a published report, apoptosis mediated through the Fas/Fas-ligand pathway was considered to contribute to the spontaneous regression of lesions in single-system LCH [20]. Limited information is available on HP-LCH therapy, and the treatment of isolated HP-LCH is controversial and individualized. Only a few small-sample-size studies or case reports have been published [1]. Simple observation, surgery, low-dose radiation and chemotherapy are considered in the treatment planning [19]. Limited literature data show that low-dose irradiation (≤ 22 Gy) is usually the first-line treatment and adequate in most cases of isolated HPR involved LCH. Although previous studies have shown that the natural course of LCH cannot be changed by the available treatment, a few literatures have shown that radiation treatment applied in the early stages may be effective, with partial or even complete remission [21]. According to current standard therapy in children, total surgical excision achieves good efficacy only in treating isolated CDI patients. However, post-surgical recurrence is observed in some studies [1]. Horiguchi et al. reported a case of recurrence after tumorectomy of an isolated HP-LCH adult patient. In this article, the 53-year-old female, with a mass projecting from the hypothalamus into the third ventricle, received a total tumor resection. Three years later, tumor regrowth has been found [22]. Another case described a 50-year-old woman with LCH restricted in the sellar region firstly received gamma knife radiosurgery after diagnosed. However, 5 years later, MRI indicated tumor recurrence in the sellar region. Subsequently, tumorectomy was performed. One year after surgery, the nodular shadow became larger, and was again considered as recurrence [23]. These cases show that recurrence may occur after either tumorectomy or radiosurgery. Haupt et al. reported a 14-year-old girl and a 9-year-old girl with isolated HP-LCH. They underwent open craniotomy and low-dose local irradiation of 20 Gy. During follow-up, their manifestations were stable without de novo lesion [24]. Another case presented a patient (a 31-year-old woman) of localized HPR involved LCH with dominant APD but without CDI. Seven years after surgery and local radiotherapy, she is stable. These cases indicate that surgery combined with low-dose irradiation may be another effective treatment for isolated HPR involved LCH [11]. In a study of assessing the activity and tolerability of 2-chlorodeoxyadenosine (2-CDA) in treating mass lesions of the HP-LCH, 2 of the 12 patients were isolated HP-LCH. They both had a sustained radiographic response to 2-CDA with no significant toxicity [25].

In our case, a male adolescent of HP-LCH presented as CDI, which is the hallmark of HPR infiltration, is rare in children and adolescents, since up to 50% of CDI are idiopathic [26]. In children with isolated HPR lesions, the differential diagnosis includes LCH, germ cell tumor and LYH [27]. Clinico-radiological evidence is beneficial in establishing hypothesis. The gold standard for LCH is positive histology and immunohistochemistry evidence. It is now widely accepted that the LCH diagnosis is divided into three steps: histology-based diagnosis, diagnosis based on histology and active S100 in lesional cells, and a definitive diagnosis established by histology and the lesional cells expressing CD1a or having intracytoplasmic Birbeck granules [28].

LYH, a neuroendocrine disease, is characterized by autoimmune monoclonal lymphocytic infiltration of the pituitary gland. Its differential diagnosis is dependent on the evidence of lymphoplasmacytic infiltration in the pituitary gland, and detection of anti-pituitary antibodies and anti-hypothalamus antibodies by indirect immunofluorescence [12]. The gold standard for diagnosis of LYH still relies on histopathological examination. LYH can be divided into two categories, namely, lymphocytic adenohypophysitis (LAH) and lymphocytic infundibuloneurohypophysitis (LINH). A majority of LAH are characterized by APD (such as GH deficiency, secondary hypothyroidism, hypogonadism, and hyperprolactinemia), while a few of LAH present as the absence of posterior pituitary hormones. GH deficiency is found in 3/4 of minor cases. LINH involving the infundibulum, pituitary stalk and neurohypophysis, triggers CDI all the time. In LINH, the typical MRI shows a diffuse thickening of the pituitary stalk. A “loss of bright spot” of the neurohypophyseal (the lack of the physiologic hyperintense signal of the posterior pituitary on T1WI) is also common [1, 29]. However, in HPR involved LCH, MRI frequently shows the enlargement of the pituitary stalk or the lack of the physiologic hyperintensity signal of the posterior pituitary on T1WI, which is hard to distinguish from LYH [1].

In conclusion, our case indicates that when an adolescent with CDI accompanied with nodular thickening of the pituitary stalk, and the absence of a high intensity signal of the neurohypophysis in MRI, not only LYH or germinoma but also LCH should be considered. Moreover, pathologic biopsy should be immediately performed. Since a patient with a single-system LCH often carries a good prognosis and isolated HP-LCH lesions frequently shrink spontaneously, pathologic biopsy and simple watchful observation may be alternative to isolated HP-LCH.

## Data Availability

The datasets supporting the conclusions of this article is included within the article and in figures.

## Abbreviations

2-CDA: 2-chlorodeoxyadenosine
APD: Anterior pituitary deficiencies
CD: Cluster of differentiation
CDI: Central diabetes insipidus
CK: Cell keratin
CNS: Central nervous system
CT: Computed tomography
DDAVP: 1-deamino-8-D-arginine vasopressin
GCTs: Germ cell tumors
GH: Growth hormone
HCG: Human chorionic gonadotropin
HPR: Hypothalamic pituitary region
IgG: Immunoglobulin G
LAH: Lymphocytic adenohypophysitis
LCH: Langerhans cell histiocytosis
LINH: Lymphocytic infundibuloneurohypophysitis
LYH: Lymphocytic hypophysitis
MRI: Magnetic resonance imaging
OCT-4: Octamer-binding transcription factor 4
PLAP: Placental alkaline phosphatase
SF1: Steroidogenic factor 1
T1WI: T1-weighted image
T2WI: T2-weighted image
